# Diverse supramolecular structures formed by self‐assembling proteins of the *B* *acillus subtilis* spore coat

**DOI:** 10.1111/mmi.13030

**Published:** 2015-05-15

**Authors:** Shuo Jiang, Qiang Wan, Daniela Krajcikova, Jilin Tang, Svetomir B. Tzokov, Imrich Barak, Per A. Bullough

**Affiliations:** ^1^ Krebs Institute for Biomolecular Research, Department of Molecular Biology and BiotechnologyUniversity of SheffieldSheffieldS10 2TNUK; ^2^ Institute of Molecular BiologySlovak Academy of SciencesDubravska cesta 21Bratislava845 51Slovakia; ^3^ State Key Laboratory of Electroanalytical Chemistry, Changchun Institute of Applied ChemistryChinese Academy of SciencesChangchun130022China

## Abstract

Bacterial spores (endospores), such as those of the pathogens *C* *lostridium difficile* and *B* *acillus anthracis*, are uniquely stable cell forms, highly resistant to harsh environmental insults. *B* *acillus subtilis* is the best studied spore‐former and we have used it to address the question of how the spore coat is assembled from multiple components to form a robust, protective superstructure. *B* *. subtilis* coat proteins (CotY, CotE, CotV and CotW) expressed in *E* *scherichia coli* can arrange intracellularly into highly stable macro‐structures through processes of self‐assembly. Using electron microscopy, we demonstrate the capacity of these proteins to generate ordered one‐dimensional fibres, two‐dimensional sheets and three‐dimensional stacks. In one case (CotY), the high degree of order favours strong, cooperative *intracellular* disulfide cross‐linking. Assemblies of this kind could form exquisitely adapted building blocks for higher‐order assembly across all spore‐formers. These physically robust arrayed units could also have novel applications in nano‐biotechnology processes.

## Introduction

Cellular development involves the formation of complex supramolecular structures from many components. An essential characteristic of many such processes is self‐assembly in which components spontaneously form ordered aggregates (Whitesides, 2002; Whitesides and Boncheva, 2002). However, for complex higher‐order structures, there have to be instructions that direct the components to the right spatial locations, determined through a combination of the intrinsic structures of the proteins themselves and other cellular factors. As an example, different chemical and physical characteristics of cell membranes (e.g. membrane curvature) determine localisation of some proteins in bacterial cells (Barák and Muchová, 2013). A well‐studied model for cellular assembly is the differentiated cell‐form of the bacterium *Bacillus subtilis* – the endospore – that is specialised to survive environmental stress (Henriques and Moran, 2007; McKenney *et al*., 2012).

The underlying principles governing endospore assembly are probably similar in distantly related *Bacilli* and *Clostridia* species, but the molecular details are poorly understood (Giorno *et al*., 2007; Saujet *et al*., 2013). However, across species, fully assembled endospores demonstrate astonishing longevity and resilience to environmental insults; much of this resilience is conferred by the exterior protein coat (Nicholson *et al*., 2000; Klobutcher *et al*., 2006; Setlow, 2006; Henriques and Moran, 2007; Laaberki and Dworkin, 2008). In the case of *B. subtilis*, the coat uses an astonishing 70 or more different proteins (Henriques and Moran, 2007; McKenney *et al*., 2012) to build a multilayered structure – this is an assembly problem an order of magnitude more complex than a typical virus coat for example (King, 1980). Thus investigation of the mechanisms of coat assembly could reveal new principles in the construction of highly complex cellular structures.

The first clear morphological feature of sporulation is the formation of an asymmetric septum, which divides the cell into a larger mother cell and a smaller forespore. In the next stage of development the mother cell engulfs the forespore. The proteins of the spore coat are synthesised within the mother cell and subsequently deposited on the developing surface of the forespore in a highly regulated manner (Driks *et al*., 1994). In *B. subtilis*, four major coat zones have been identified: the basement layer, inner coat, outer coat and crust (Driks, 1999; Henriques and Moran, 2000; 2007; Kim *et al*., 2006; McKenney *et al*., 2010; 2012; Imamura *et al*., 2011). The relative positioning of many individual proteins within these layers has been established through a spatial interaction map (Kim *et al*., 2006; McKenney *et al*., 2010). A study of timing of protein deposition on the spore surface showed that coat assembly starts at one pole of the spore (Wang *et al*., 2009; McKenney and Eichenberger, 2011; Setlow, 2011) and involves proteins from all four layers, with assembly proceeding in several waves. Late‐expressed proteins are likely to be added by diffusion through the permeable coat layers although eventually the coat becomes impermeable (McKenney *et al*., 2012) except to small molecules such as germinants. ‘Morphogenetic proteins’ are key to organising the assembly of individual coat layers: SpoIVA for the basement layer, SafA for the inner coat, CotE for the outer coat and CotZ for the crust (Driks *et al*., 1994; McKenney *et al*., 2012). The central importance of these factors is illustrated by the example of a *spoIVA* mutant in which forespores are coatless, but coat protein aggregates are observed in the mother cell cytoplasm (Coote, 1972; Piggot and Coote, 1976; Roels *et al*., 1992). These aggregates do have some of the layered structure seen in intact wild‐type coats, hinting at a capacity for self‐assembly of at least some of the coat components. The structural details of the coat architecture and the role of the morphogenetic proteins in determining this architecture have yet to be revealed at the molecular level, but formation of multimers seems to play a key role. For example, purified SpoIVA polymerises into cable‐like structures in an adenosine triphosphate‐dependent manner (Ramamurthi and Losick, 2008), and the other morphogenetic proteins SafA, CotE and CotZ also show homotypic interactions (Little and Driks, 2001; Ozin *et al*., 2001; Krajcikova *et al*., 2009).

Different protein–protein interactions have been identified among the coat components (reviewed in McKenney *et al*., 2012). In this paper, we explore some of these interactions through observation of heterologously expressed coat proteins. This continues from the work of Krajcikova *et al*. (2009) who probed coat proteins from the so‐called ‘insoluble fraction’ – CotE, CotV, CotW, CotX, CotY and CotZ. These are all proteins associated with the outer coat and crust (Zhang *et al*., 1993). During sporulation, these proteins are among the most highly expressed of all the *B. subtilis* proteins (Mäder *et al*., 2012; Nicolas *et al*., 2012). CotE orthologues are found across a broad range of *Bacillus* species, including those of the *Bacillus cereus* group (Henriques and Moran, 2007). Orthologues of CotV–CotZ do not appear to be found in all *Bacillus* species, but are nevertheless widely distributed; notably CotY appears to be common to all members of the *B. cereus* group as well as *B. subtilis*. None of the proteins mentioned earlier appears among the genomes of *Clostridia* (Henriques and Moran, 2007). In *B. subtilis*, genes encoding CotV and CotW lie upstream of the *cotXYZ* operon and are grouped in one gene cluster, implying some functional relationship (Zhang *et al*., 1993). CotY and CotZ are cysteine‐rich proteins with CotY containing 15 cysteines (out of 161 residues) (Zhang *et al*., 1993; Kuwana *et al*., 2002). CotY is present in multimeric forms (Zhang *et al*., 1993). Krajcikova *et al*. identified *in vitro* interactions between CotY and CotZ, CotV and CotW, along with homotypic interactions of CotE and CotY. Following from this work, we now identify some key questions that we can start to address by analysing interactions among these coat proteins in more detail:

(i)  To what extent does the architecture of the coat arise from self‐organisation?
(ii)  How is the predicted protein interaction network (Krajcikova *et al*., 2009; McKenney *et al*., 2010) reflected in the three‐dimensional architecture of coat assemblies?
(iii)  What are the specific interactions that position Cot proteins in the coat and what stabilises them?
(iv)  How are the characteristic ‘shell‐like’ structures formed?
(v)  Are there common features of coat assembly across bacterial species?

In the present study, we do not attempt to answer all these questions fully – that will require a combination of cell biology, genetic and biochemical approaches. However, our analysis of coat protein macro‐structures does shed light on all of them.

## Results

### Heterologous expression and purification of CotY

**Figure 1:**
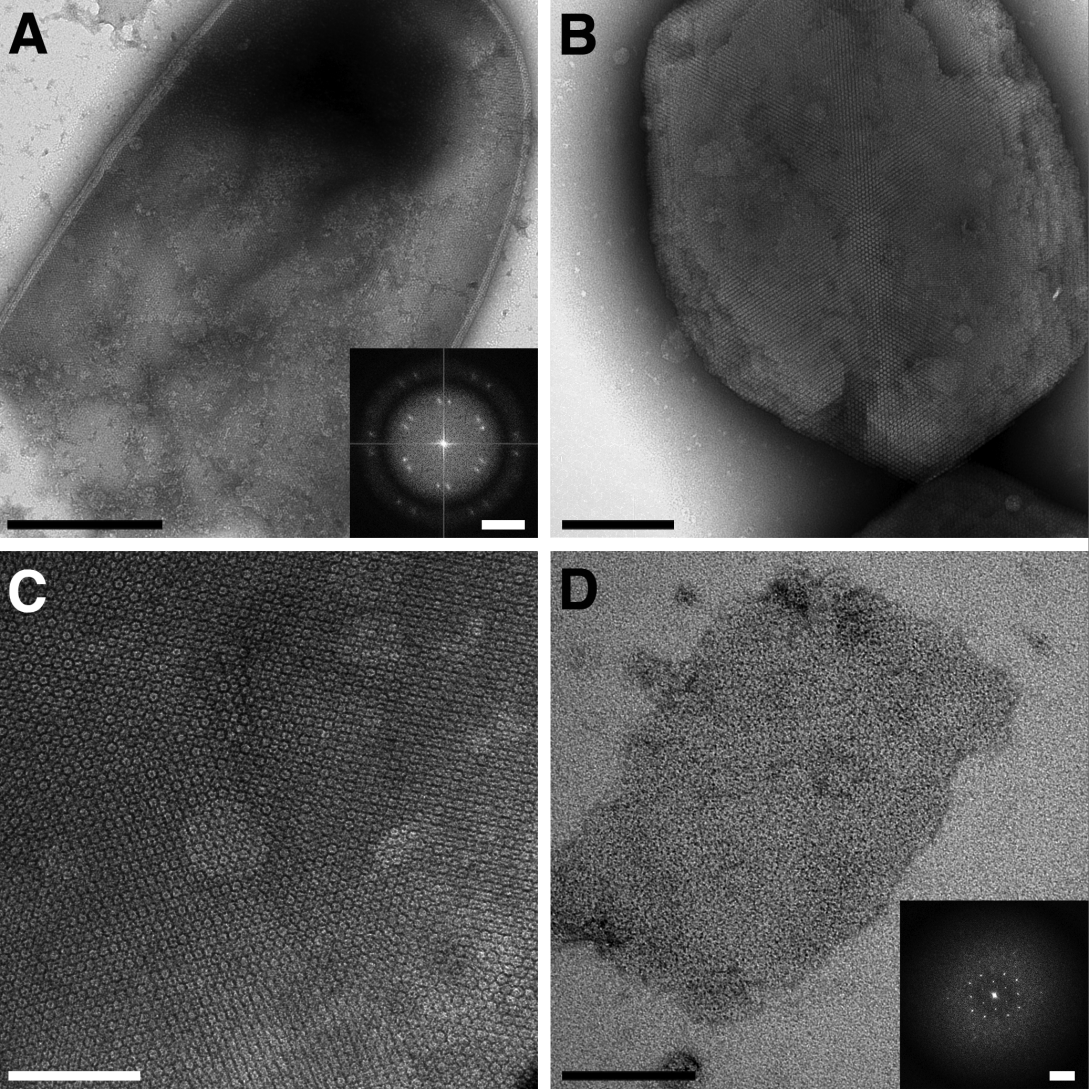
Two‐dimensional crystals of CotY. A. Negatively stained image of a broken *E* *scherichia coli* cell after induction to express His_6_‐CotYc followed by sonication. Scale bar represents 500 nm. Inset shows a computed diffraction pattern from an area within the cell image. Scale bar represents 0.14 nm^−1^. B. Negatively stained multilayered His_6_‐CotY crystal released from a sonicated *E* *. coli* cell. Crystals were obtained from two independent cell batches. Scale bar represents 200 nm. C. Magnified image of His_6_‐CotY crystal showing Moiré pattern. Scale bar represents 100 nm. D. Thin negatively stained His_6_‐CotYc crystal released from a sonicated *E. coli* cell. Crystals were obtained from two independent cell batches. Scale bar represents 100 nm. Inset shows a computed diffraction pattern. Scale bar represents 0.28 nm^−1^.

**Figure 2:**
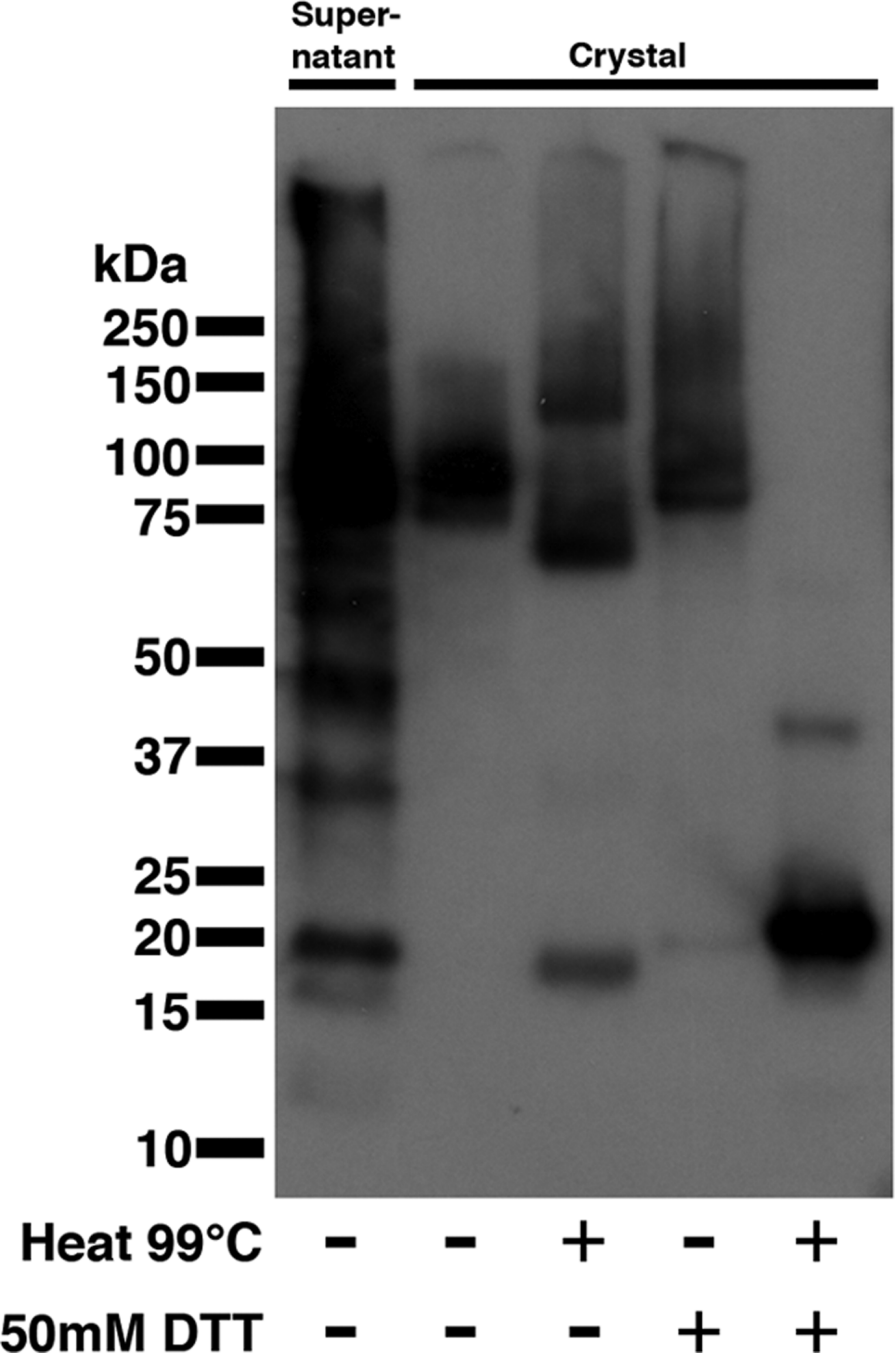
Western blot analysis of purified His_6_‐CotY crystal and supernatant fractions from *E* *scherichia coli* overexpression. Lane 1 contains untreated nickel‐affinity batch purified supernatant fraction. Lanes 2–5 show nickel‐affinity batch purified CotY crystals treated with combinations of denaturing conditions: heating at 99°C and 50 mM DTT over 20 min. Bands present at the top of lanes 2–5 indicate material trapped at the well interface. The recovered yield of crystals in the batch purification is not known, so protein signal in lane 1 cannot be compared with that in the ‘crystal’ lanes.

*Escherichia coli* cells induced to express N‐terminally poly‐histidine tagged CotY (His_6_‐CotY) were disrupted by sonication and the samples examined by electron microscopy (EM). Remarkably, we found partially broken cells densely packed with crystalline material that yielded diffraction spots in Fourier transforms (Fig. 1A). We also found a large number of extensive crystalline sheets liberated from the cells, both in the presence and absence of 8 M urea (Fig. 1B and C). These crystals often appeared to consist of several stacked layers (Fig. 1B). However, we discovered that the addition of an extra peptide segment at the C‐terminus yielded thinner crystalline sheets of a generally uniform thickness (Fig. 1D). For this extended construct, cloning of the *cotY* gene into the pET28a plasmid introduced 19 amino acid residues in position 176–201 of the CotY C‐terminus while removing residues 176–181 (Fig. S1). We denote this construct His_6_‐CotYc [predicted molecular weight (MW) ∼22.2 kDa]. For the His_6_‐CotY crystals, we used batch purification in which crystals were adsorbed to NiNTA‐agarose beads and eluted using imidazole/urea buffer, followed by centrifugation and resuspension. We tested the stability of the crystals to detergent, reducing agents and heat and examined them by EM, SDS‐PAGE and Western blotting (Fig. 2, Fig. S2). Crystals were found to be largely insoluble in 1% SDS with only a faint streak of material being visible by SDS‐PAGE at an MW corresponding to > 80 kDa (Fig. S2, lane 2). This streak was confirmed by Western blot analysis, which showed the presence of CotY at ∼ 80 kDa and larger (Fig. 2, lane 2). By contrast, the supernatant showed a more distinct staining at an apparent MW of ∼ 19 kDa, corresponding to His_6_‐CotY along with faint, but distinct soluble CotY multimers (Fig. S2, lane 1), which were confirmed by Western blotting (Fig. 2, lane 1). Heating the crystals to 99°C for 20 min mildly disrupted the crystal with a faint band visible at ∼ 19 kDa and a faint shifted band at ∼ 75 kDa on SDS‐PAGE (Fig. S2, lane 3). Western blot analysis confirmed the presence of a ∼19 kDa monomer and high MW complexes with two smeared bands at ∼75 kDa and ∼140 kDa (Fig. 2, lane 3). Incubation at room temperature in 50 mM dithiothreitol (DTT) for 20 min did not significantly reduce the population of ordered crystals as judged by EM but some material of apparent MW ∼ 80 kDa and larger did become visible by SDS‐PAGE (Fig. S2, lane 4). Western blotting confirmed the presence of complexes ∼ 80 kDa and above (Fig. 2, lane 4). After incubation in 50 mM DTT for 20 mins at 99°C, no ordered crystals were visible; instead we found amorphous aggregates and small particles of diameter ∼ 70 Å with only a ∼19 kDa band visible by SDS‐PAGE (Fig. S2, lane 5). Western blot analysis confirmed the presence of a strong monomeric ∼ 19 kDa band and faint putative dimer at ∼ 40 kDa (Fig. 2, lane 5). It is essential to note that only under heat and reducing conditions did the high MW complexes, which were also detected in the wells of the SDS‐PAGE gel, disappear completely (see the bands at the top of Fig. 2). Thus, a strong correlation between the disassembly of crystals with the appearance of monomeric CotY can be drawn through both SDS‐PAGE and Western blot analysis.

**Figure 3:**
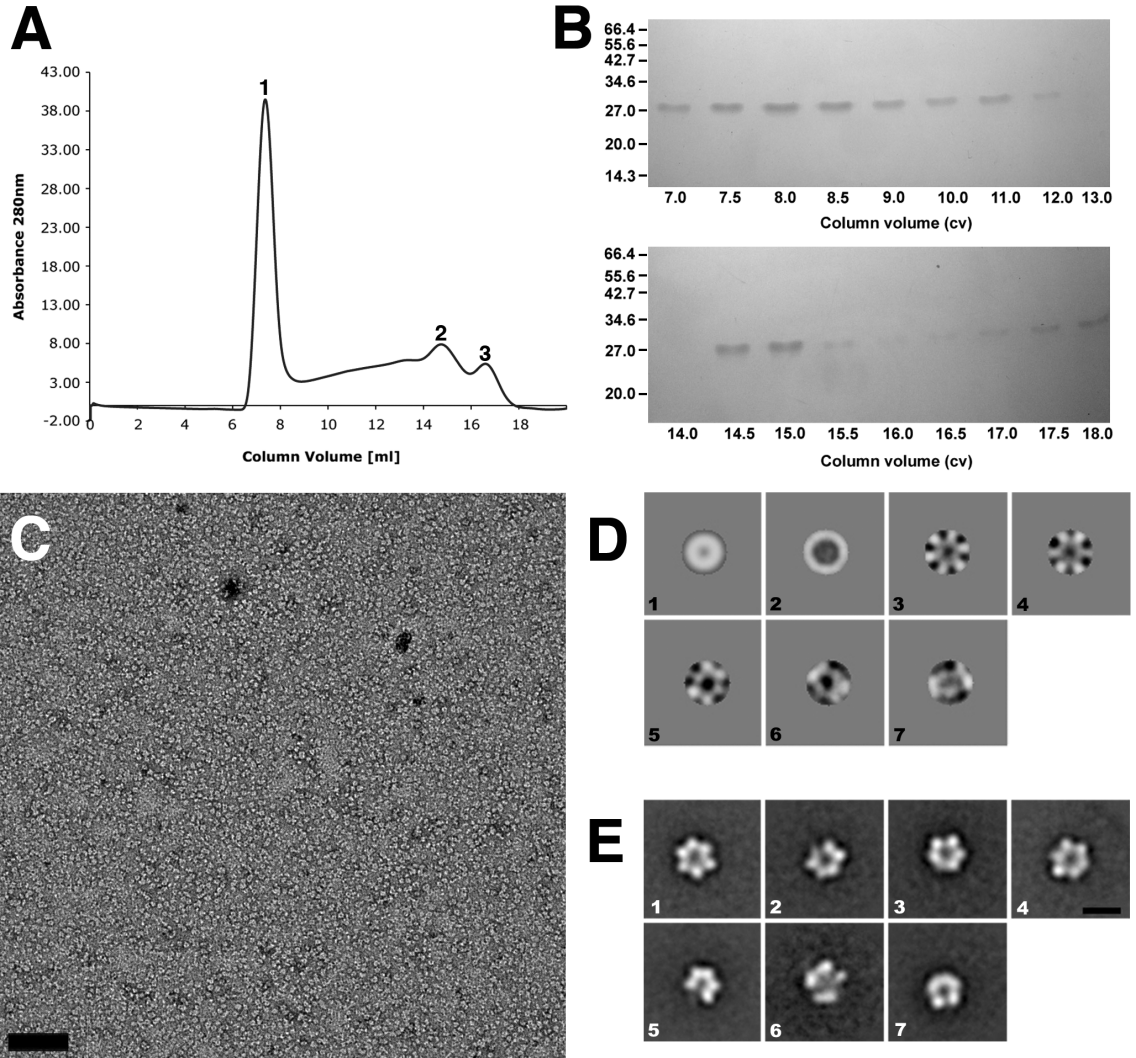
Hexameric particles isolated using size‐exclusion chromatography and single particle averaging. A. Elution profile from size‐exclusion chromatography of His_6_‐CotYc after nickel‐affinity purification with three major peaks labelled 1, 2 and 3 representing about 50%, 16% and 6% of the total eluted protein, respectively. B. SDS‐PAGE of eluted fractions from size‐exclusion chromatography. C. Negatively stained image of monodisperse His_6_‐CotYc hexameric particles from fraction 3 (panel A). Scale bar represents 100 nm. Particles were obtained after protein purification from two independent cell batches. D. Eigenimages from MSA; [1] is the rotational average of the particles; [2] shows the diameter variation; [3–5] indicate sixfold symmetry. E. His_6_‐CotYc class averages. Scale bar represents 10 nm.

The His_6_‐CotYc construct yielded a more soluble product that we subjected to both nickel‐affinity and size‐exclusion chromatography (Fig. 3A). The elution profile from size‐exclusion chromatography exhibited three major protein peaks: peak 1 corresponded to the void volume; the very broad peak 2 corresponded to an apparent MW of 340 kDa and upwards; peak 3 corresponded to an apparent MW of 115 kDa (equivalent to the predicted MW of a pentamer or hexamer). The eluted fractions analysed by SDS‐PAGE indicated a protein species of ∼ 28 kDa (Fig. 3B) (expected, 22.2 kDa). Each fraction was interrogated by EM. Peak 1 fractions yielded amorphous aggregates and some large, well‐ordered crystalline sheets up to 0.4 μm on a side – these were similar to crystals released directly from sonicated *E. coli* cells (Fig. 1D). Peak 2 fractions revealed a small number of crystalline patches and protein aggregates. Peak 3 showed a largely monodisperse sample of single particles with an apparent diameter of ∼ 100 Å (Fig. 3C).

**Figure 4:**
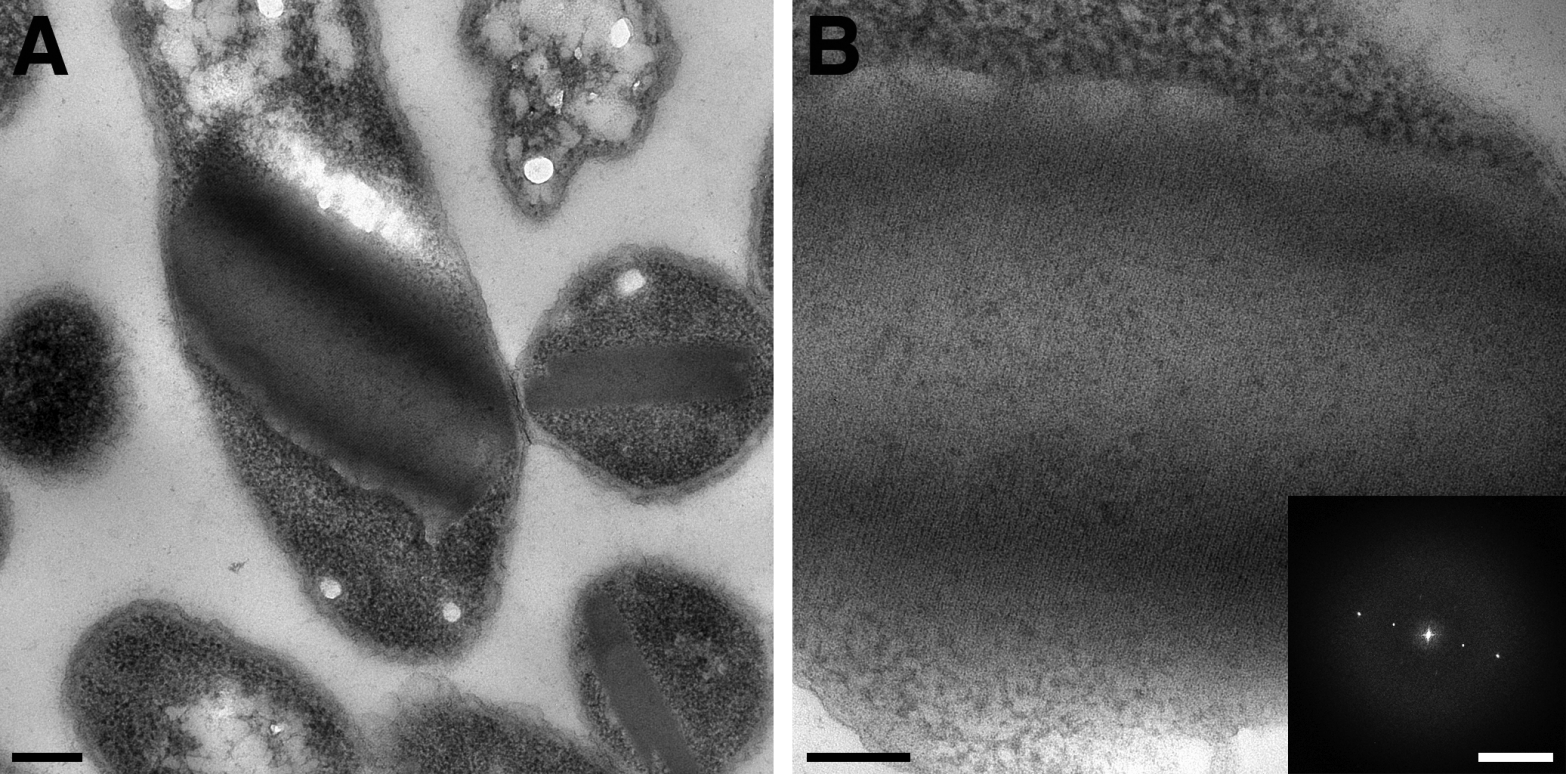
Thin sections of *E* *scherichia coli* overexpressing CotY multi‐layered crystals. A. Large self‐assembled intracellular CotY crystals within *E* *. coli* BL21(DE3) pLysS. Scale bar represents 0.2 μm. B. Magnified thin section through a multi‐layered CotY crystal. Scale bar represents 100 nm. Inset shows a computed diffraction pattern from within the image. Scale bar represents 0.35 nm^−1^.

In addition to His_6_‐CotY and the His_6_‐CotYc variant, we prepared recombinant untagged CotY. Importantly, all three variants were crucial for understanding the mode of self‐assembly of CotY. The untagged CotY protein was not amenable to batch purification for rigorous biochemical analysis, but large crystalline sheets could be released from broken cells and appeared similar to those of the His_6_‐CotY and His_6_‐CotYc proteins (Fig. 1C and D), including identical unit cell parameters and plane group symmetry (see later). Electron micrographs of thin sections of cells expressing CotY often revealed the stacking of multiple crystalline layers, with a fundamental spacing of ∼ 63 Å between layers. However, the strong second‐order diffraction spot also revealed strong contrast at a spacing of ∼ 63/2 Å (Fig. 4, inset) indicating the presence of sublayers with ∼ 32 Å spacing.

### His_6_‐CotYc single particle image analysis

Images of 2278 particles from peak 3 (Fig. 3A and C) were selected for multivariate statistical analysis (MSA; van Heel *et al*., 1996). Examination of the main components (eigenimages) contributing to the images provided clear evidence of sixfold rotational symmetry (Fig. 3D). Seven class averages obtained after five rounds of multi‐reference alignment (MRA) are shown in Fig. 3E; the class average in panel 1 indicates a ring‐like structure with a central stain‐filled cavity or hole and six subunits. This is entirely consistent with the approximately hexameric assembly state predicted from peak 3 of the size‐exclusion profile (Fig. 3A). The class averages in the remaining panels are likely to represent similar particles that are either slightly tilted and/or have one or more subunits missing.

### CotY‐crystallographic image analysis

**Figure 5:**
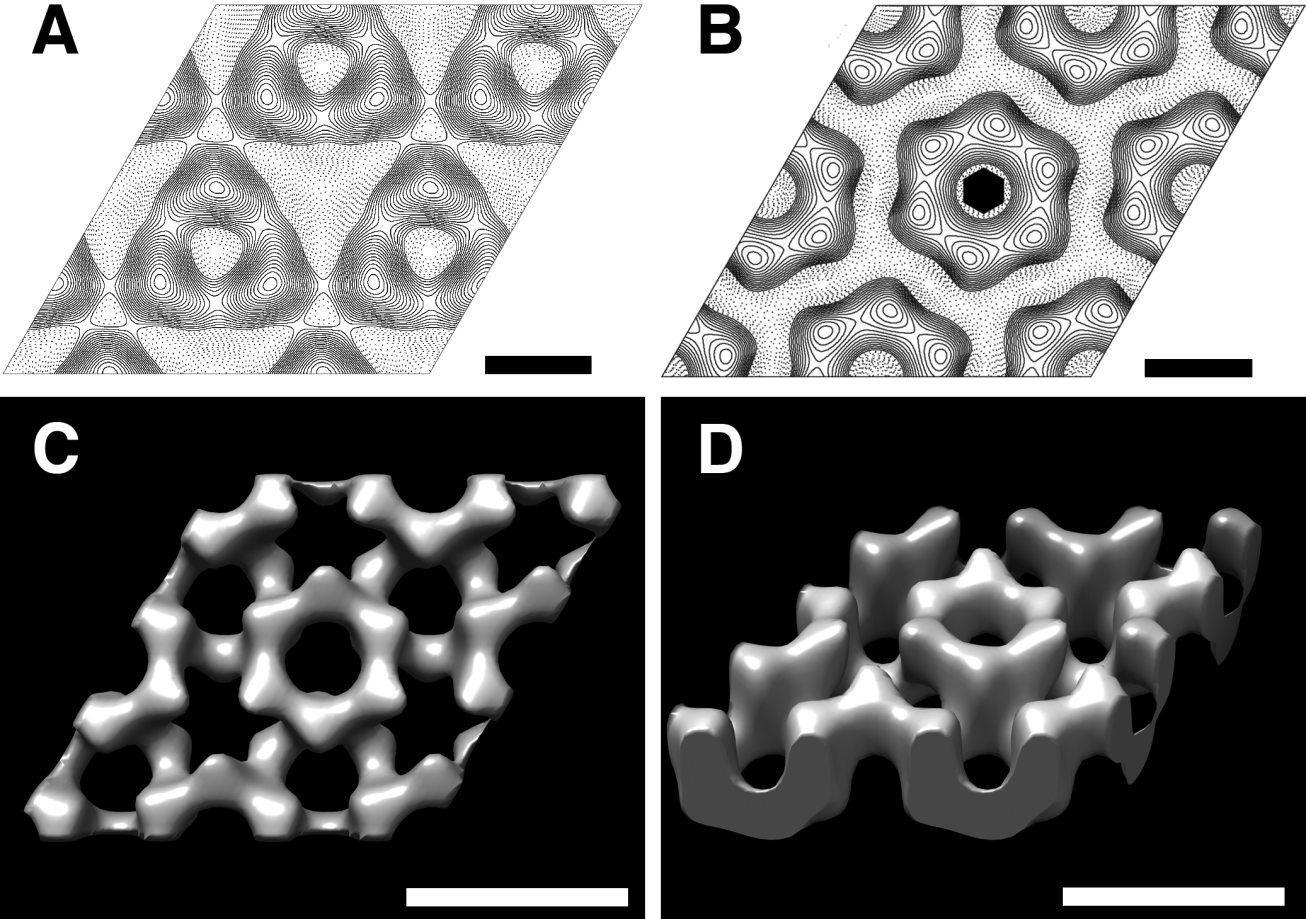
Projection maps and three‐dimensional reconstruction of His_6_‐CotYc crystals. A. Averaged contoured projection map of negatively stained His_6_‐CotYc two‐layered crystal with threefold (*p*3) symmetry enforced. Solid contours represent density below the mean (i.e. stain‐excluding). Scale bars represent 43 Å. B. Projection map calculated from one hexagonal form crystal with sixfold (*p*6) symmetry imposed. Contours as in (A). The hexagon symbol represents the crystallographic sixfold symmetry axis. Scale bars represent 43 Å. C and D. Three‐dimensional surface representations of negatively stained two‐layered crystal form with *p*3 symmetry enforced. Dark regions represent highly stained parts of the crystal. Scale bars represent 86 Å.

The full‐length (CotY), polyhistidine‐tagged (His_6_‐CotY) and extended forms (His_6_‐CotYc) of CotY all yielded crystals that had identical unit cell parameters (within experimental uncertainty). However, we focused our image analysis on the crystals of the extended construct His_6_‐CotYc (Fig. 1D) as this gave thinner crystals of generally uniform thickness – unlike the variably layered CotY and His_6_‐CotY crystals. These variably layered crystals were not amenable to three‐dimensional image analysis because data can only be merged from multiple independent images if they are from crystals that all have an identical number of layers. For His_6_‐CotYc crystals, the mean unit cell parameters were *a* = *b* = 86.5 ± 1.5 Å, *γ* = 120 ± 1° (*n* = 20). For all samples, phases were consistent with *p*3 (threefold) symmetry and a subset was also consistent with *p*321 (threefold with perpendicular twofold) (Table S1); a projection map is shown in Fig. 5A, with *p*3 symmetry enforced. In one sample only, phase comparisons indicated *p*6 (sixfold) or *p*622 (sixfold with perpendicular twofolds) symmetry (Table S2); the projection map from this one sample, with *p*6 symmetry enforced, (Fig. 5B) exhibits a lattice of rings, each consisting of six prominent stain‐excluding densities. The ring has a densely stained core (marked by the hexagon symbol in Fig. 5B). This projection is qualitatively similar to that of the hexameric single particles in Fig. 3E, panel 1, although the ring has a slightly smaller diameter than that measured from single particles (Fig. 3E); this may reflect uncertainty in determining the stain boundary in single particle images, or possibly a different conformation of the hexameric assembly.

We merged the data for *p*3/*p*321 crystal forms and subjected them to three‐dimensional analysis, but enforcing only *p*3 symmetry. Eight tilt series were recorded up to tilt angles of 55° with 113 images contributing to the final structure. The distributions of phase and amplitude measurements along z* are shown in Fig. S3. A representation of the calculated three‐dimensional density distribution is shown in Fig. 5C and D (see also Movie S1). The reconstructed density appears as two layers of hexameric rings, staggered relative to each other by half a unit cell in each direction (**a**/2 + **b**/2). The projection of a single layer from this double‐layered assembly would be similar to that shown in Fig. 5B, suggesting that this rare form corresponds to a single layer of CotY hexamers, with *p*6 symmetry. However, it could also be a double layer with rings opposing, but in register to give *p*622 symmetry. In the *p*3 two‐layer form (Fig. 5C and D), the staggered layers superimpose to yield the projection shown in Fig. 5A. The threshold level for surface contouring in Fig. 5C and D was set to the midpoint of the maximum density gradient; this encloses a volume corresponding to ∼ 60 kDa per hexameric ring (∼ 10 kDa per subunit), assuming a protein density of 1.3 g cm^−3^. However, it is possible that the true density is higher than this. The total depth of the two layers is ∼ 60 Å.

### CotV–CotW – EM and atomic force microscopy (AFM)

**Figure 6:**
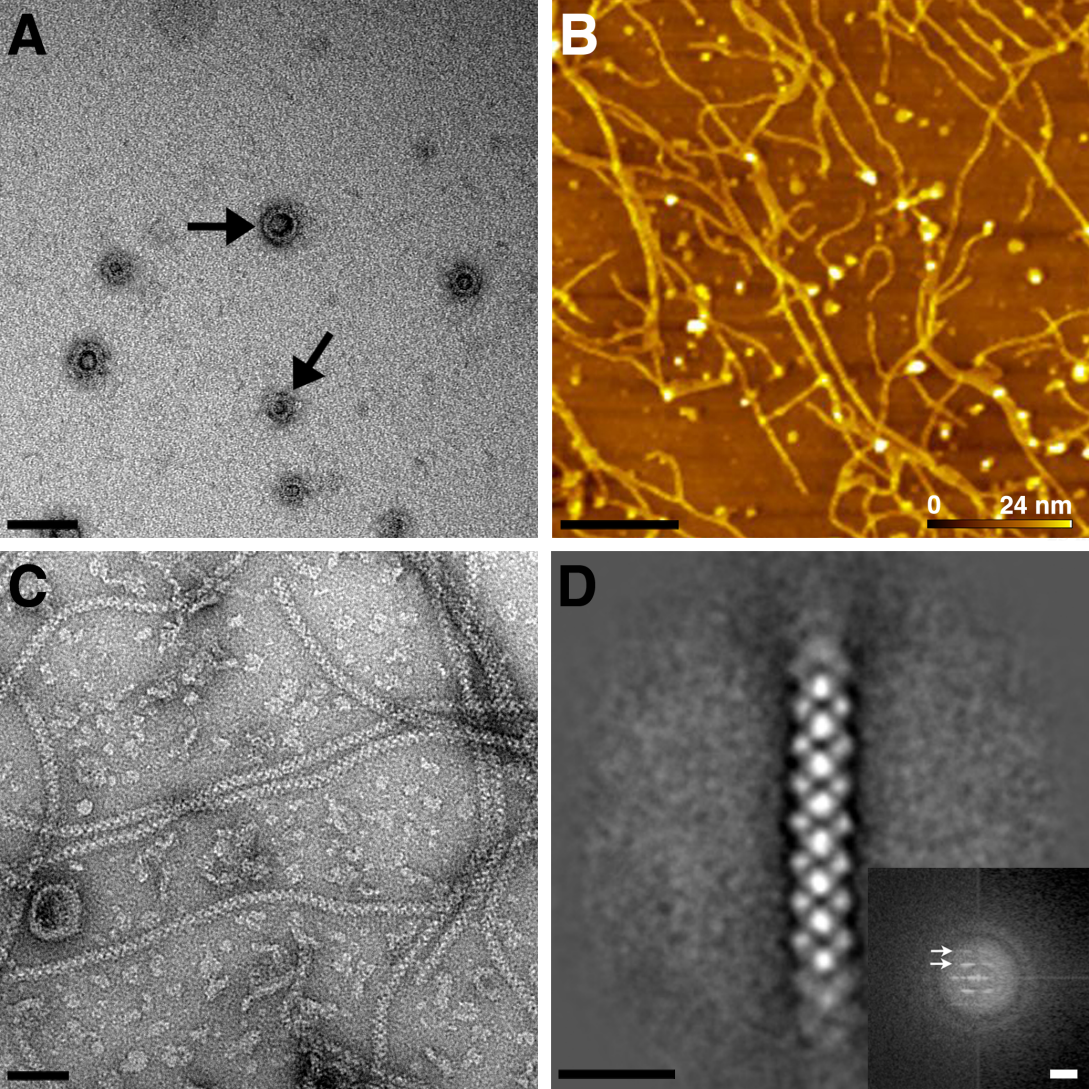
EM and AFM of CotV–W. A. Negatively stained image of CotW particles after *E* *scherichia coli* overexpression and purification. Scale bar represents 100 nm. B. AFM topograph of CotV–CotW on a silicon surface. Heights above the surface are colour coded. Scale bar represents 1 μm. C. Electron micrograph of negatively stained CotV–CotW co‐expression showing helical fibres. Scale bar represents 50 nm. D. Single‐particle average of 137 fibre segments. Scale bar represents 20 nm. Inset shows computed diffraction from raw image (right) and averaged image (left). Arrows indicate layer lines. Scale bar represents 0.3 nm^−1^. Images (B) and (C) were obtained after protein purification from two independent cell batches.

The soluble complex of CotV–CotW extracted from a strain expressing both proteins was purified using nickel‐affinity chromatography targeted at a hexahistidine‐tag at the N‐terminus of CotV. CotW was co‐purified via contact with CotV as seen in Fig. S4. Western blot analysis confirms the presence of both proteins in the purified complex (Krajcikova *et al*., 2009). We were not able to use EM to inspect CotV expressed on its own because of the very low yield of soluble protein. However, we could detect CotV by Western blotting (Krajcikova *et al*., 2009). By contrast, CotW expressed on its own was sufficiently soluble for EM analysis. In this case, particles of CotW appeared in projection as a central stain – excluding density encircled by a stain‐filled moat and an outer stain‐excluding ring. The particles varied from 200 Å to 700 Å in diameter (Fig. 6A). However, these characteristic particles were rare in images of co‐expressed CotV–CotW preparations (Fig. 6C). Instead, the most striking feature was a network of tangled fibres purified from the *E. coli* host, with a background of more amorphous material (Fig. 6B and C). The projected fibres viewed by EM were consistently of ∼ 100 Å diameter (Fig. 6C). The height of the fibres estimated by AFM (Fig. 6B) was similar, indicating that the fibres have a cylindrical cross section. The right half of the inset in Fig. 6D shows a Fourier transform computed from a selected length of one of the straighter fibres in an electron micrograph, while Fig. 6D main panel shows an averaged image calculated from 137 fibre sections; the Fourier transform of this average is shown in the left hand panel of the inset, showing qualitatively similar layer line features to the right half. Both transforms indicate an axial repeat along the fibre of ∼67 Å.

### CotE‐purification and EM

**Figure 7:**
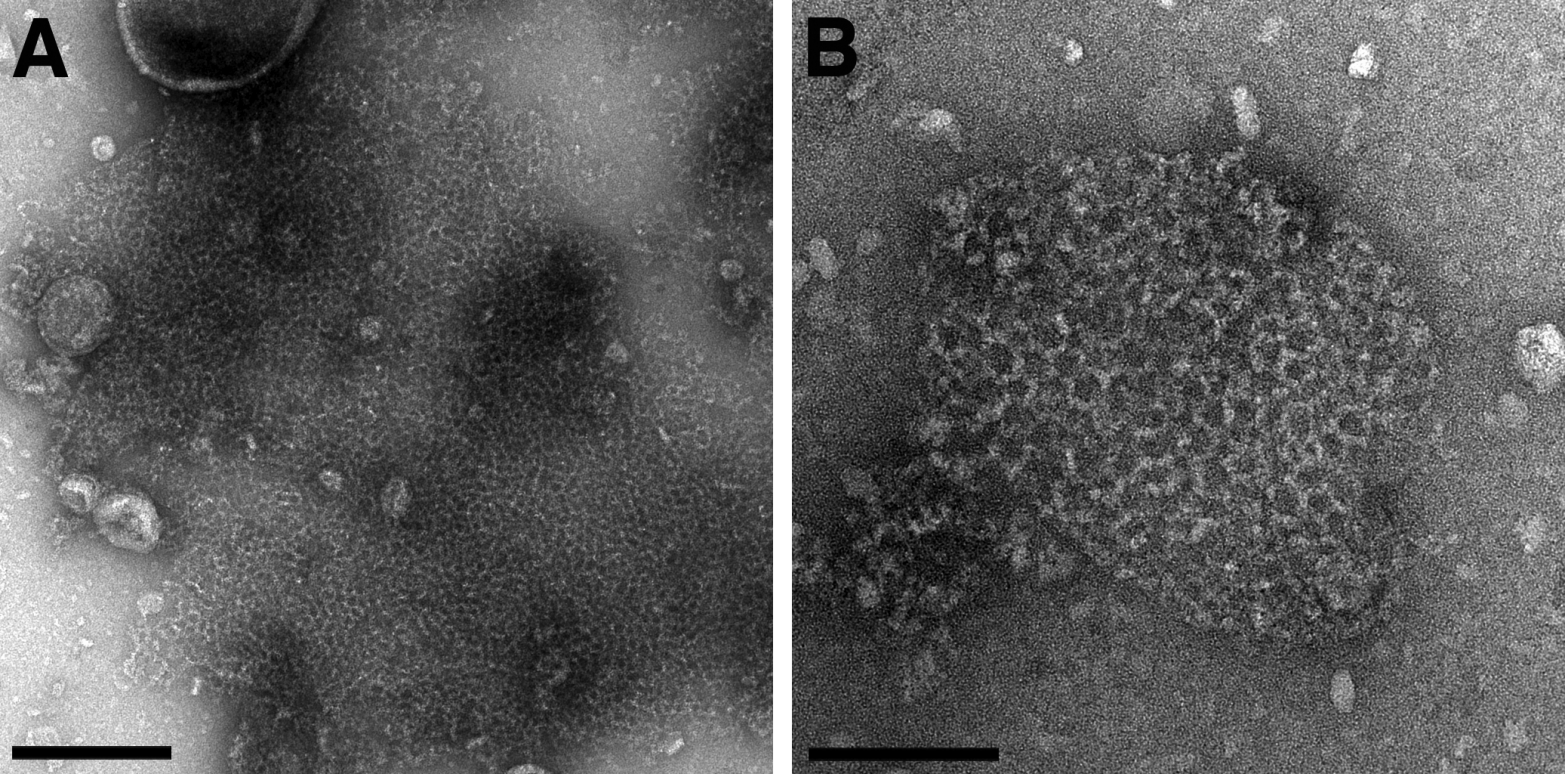
Two‐dimensional quasi‐crystals of CotE. A. Negatively stained image of broken *E* *scherichia coli* cell after CotE expression followed by sonication. Scale bar represents 100 nm. B. Magnified image of CotE quasi‐crystal. Scale bar represents 200 nm. Quasi‐crystals were obtained from five independent cell batches.

CotE was largely insoluble, but aggregates could be released from sonicated/lysozyme‐treated cells. These aggregates were further enriched by a batch procedure in which they were adsorbed to NiNTA‐agarose beads and eluted using an imidazole buffer, followed by centrifugation. Fig. 7 shows electron micrographs of typical CotE aggregates displaying extended net‐like structures with an average repeat spacing of about ∼ 150 Å, although the mesh size was highly variable. The fibres of the mesh had a width of ∼ 45 Å. SDS‐PAGE analysis indicated a single species of ∼ 27 kDa (Fig. S5) close to the expected MW of 23.1 kDa. Incubation of the aggregates in 50 mM DTT or at 95°C destroyed the lattice. The N‐terminal polyhistidine tag was removed by thrombin cleavage at a site downstream of the tag and upstream of the protein; this had no effect on the net integrity.

## Discussion

The study of spore coat assembly has been challenging because solubilisation of the coat proteins has required reducing agents, detergent, alkaline pH and urea; conditions normally expected to be extremely denaturing. Nevertheless, there has been some limited success in reconstruction of the *B. cereus* coat *in vitro*, by applying coat protein extract to a spore that had been chemically stripped (Aronson and Fitz‐James, 1971); a significant role for self‐assembly is clearly evident from subsequent work (Goldman and Tipper, 1978; Aronson *et al*., 1992; Ramamurthi and Losick, 2008). To explore this process, instead of working with extracted proteins, we have focused on well‐defined, heterologously overexpressed proteins. For convenience of purification, we worked largely with polyhistidine‐tagged proteins. Where tested, these tagged proteins assembled in similar modes to those without tags.

### CotY assemblies

Our extended construct (His_6_‐CotYc) formed hexameric particles (Fig. 3C and E), which can apparently form a higher‐order assembly consisting of sheets mostly two molecules thick (Figs 1D and 5). This is entirely consistent with the prediction that CotY forms homotypic multimers in the coat (Zhang *et al*., 1993; Krajcikova *et al*., 2009). The *p*321 symmetry of a subset of the crystals would indicate a ‘head‐to‐head’ packing of the two layers. We speculate that ‘head‐to‐head’ packing could limit the thickness of the crystals to two layers and in principle this could be a general mechanism for favouring the restriction of layer thickness in the native coat. CotY and His_6_‐CotY crystals were less fragile and tended to form multilayered stacks (Figs 1B, C and 4). The basic repeat in the stacks corresponded to a thickness of ∼ 63 Å (Fig. 4), suggesting that each repeating unit was made up of two single‐molecule layers, possibly identical to the His_6_‐CotYc double layer Fig. 5C and D. Very strong second‐order diffraction in the Fourier transform (Fig. 4B inset) indicates very strong contrast at a spacing of 32 Å, consistent with the presence of a sub‐layer only one molecule thick. As the basic repeat appears to be a double layer, it is likely that head‐to‐head packing is retained in these stacks. The identical unit cell parameters and symmetry found in CotY, His_6_‐CotYc and His_6_‐CotY crystals indicate that there is no effect of the tags on structure other than the degree of fragility of the crystals and propensity for layer stacking.

The mode of assembly we see for CotY is displayed *in vivo* in at least one other bacterial group. The *p*6 crystal (Fig. 5B) resembles the *in situ* basal layer crystal of the outermost spore layer – exosporium – of members of the *B. cereus/Bacillus anthracis/Bacillus thuringiensis* group (Ball *et al*., 2008; Kailas *et al*., 2011); the possible relatedness of the *B. subtilis* spore crust (McKenney *et al*., 2010) and the *B. cereus* exosporium has been noted (Imamura *et al*., 2011). Notably, CotY has been proposed either as a component of (Imamura *et al*., 2011) or to be involved in assembly of the crust (McKenney *et al*., 2010). Furthermore, *B. cereus* exosporium contains two orthologues of *B. subtilis* CotY, namely ExsY and CotY, which each share ∼35% identity with *B. subtilis* CotY. We speculate that in the *B. cereus* group, ExsY and/or CotY naturally self‐organise *in situ* to form a two‐dimensional array similar to that of the individual single layers of *B. subtilis* CotY lattices (Fig. 5).

We have demonstrated the capacity for CotY to self‐organise into extensive ordered arrays in a non‐native, albeit cellular, environment. In the native spore it may be that long‐range extended arrays are prevented from forming through interactions with other coat proteins. It is likely that the *E. coli* overexpression system is producing more protein than in the native mother cell, but in spores, only a small surface needs to be covered. Nevertheless, it is notable that CotY is the most highly expressed of all *B. subtilis* proteins in the later stages of sporulation, with CotV and CotW not far behind (Mäder *et al*., 2012; Nicolas *et al*., 2012); it is likely that this ordered and probably highly cooperative packing is at least reflected in short‐range interactions among CotY subunits. Significantly, one or more unidentified proteins do form extensive hexagonally symmetric arrays *in situ* (Holt and Leadbetter, 1969; Aronson and Fitz‐James, 1976; Ebersold *et al*., 1981; Plomp *et al*., 2005a, 2005b, 2007). These arrays have been reported to have spacings of ∼ 90 Å, close to the 87 Å we find in our CotY assemblies. The location of this hexagonal (‘honeycomb’) layer specifically within the *B. subtilis* spore coat has been proposed in a recent AFM study (see fig. 14 of Plomp *et al*., 2014 for a summary diagram of the spore coat layers). The relatively open‐channel structures formed by CotY, if equivalent to *in vivo* structures, certainly appears capable of a filtering role that could select for small spore germinants to penetrate the spore coat, as proposed for *B. cereus* exosporium (Ball *et al*., 2008; Kailas *et al*., 2011). The other biochemically distinct coat layers would also have to play a similar role.

A number of covalent cross‐links, such as ε‐(γ‐glutamyl)‐lysil isopeptide bonds are involved in stabilising the spore architecture (Kobayashi *et al*., 1996). In the case of CotY, the highly conserved and abundant cysteines point immediately to a role for disulfide bonds in stabilising the assemblies. Indeed there is clear evidence that CotY forms disulfide‐linked multimers in the spore coat (Zhang *et al*., 1993). The disulfide bonds appeared to be exceptionally strong, with complete disassembly of crystals requiring both heat and reducing agent (Fig. 2). Notably, in Western blots of spore extracts run on non‐reducing gels, CotY (17.9 kDa) appeared to run as a 26 kDa monomer along with apparent dimers and trimers at 56 kDa and 78 kDa respectively (Zhang *et al*., 1993). This is consistent with our finding that His_6_‐CotYc appeared to run at ∼ 28 kDa under non‐reducing conditions (Fig. 3B). In reducing gels, on the other hand, His_6_‐CotY appeared in its monomeric form at ∼19 kDa, close to its predicted mass (Fig. 2). This suggests that disulfide bonds may also be involved in intra‐subunit folding.

### CotE assemblies

Homotypic interactions are predicted for CotE (Little and Driks, 2001; Krajcikova *et al*., 2009), and this is reflected in the assembly of extended two‐dimensional nets (Fig. 7). Images of lysed cells suggest that these nets have self‐organised within the host *E. coli* cell cytosol. A less regular, but nevertheless net‐like array of filaments from wild‐type spore coat extracts was observed by Aronson *et al*., but notably, not from a *cotE* deletion strain (Aronson *et al*., 1992).

CotE is required for outer spore coat formation, adopting a position at the interface between inner and outer coats (Zheng *et al*., 1988; Driks *et al*., 1994); its influence extends as far as the crust where assembly of CotX, CotZ, and CotW are also CotE‐dependent (Kim *et al*., 2006). It is tempting to speculate that the nets we observe reflect the ability of CotE to form a flexible matrix onto which other protein components could be assembled. In the earlier stages of coat assembly, such an open mesh would also facilitate the infiltration of inner coat proteins (Driks, 1999). The two‐dimensional nature of the array is ideal for the construction of a shell‐like assembly expected for CotE in the native spore coat (Driks, 1999).

### CotV–CotW assembly

Krajcikova *et al*. (2009) revealed that two proteins of the insoluble spore coat fraction, CotV and CotW are in direct contact. They also showed that CotV solubility was substantially enhanced when co‐expressed with CotW. The complex of CotV and CotW proteins self‐organised into fibrous assemblies of one consistent diameter (Fig. 6B and C). We did not observe these fibres in preparations of CotW alone suggesting that they are assembled from both proteins in a fixed stoichiometry. Western blotting indicated the presence of both proteins (Krajcikova *et al*., 2009). CotV is predicted to have a large hydrophobic domain (Zhang *et al*., 1993; Krajcikova *et al*., 2009); it is possible that much of this domain becomes buried in the fibres and/or the very hydrophilic nature of CotW (Zhang *et al*., 1993) renders the co‐complex soluble.

Fibrous structures have been isolated from spore coats by Goldman and Tipper (1978). Moreover, freeze‐etch EM revealed long fibres traversing the spore in a layer just underneath the outermost shell (Holt and Leadbetter, 1969; Aronson and Fitz‐James, 1976). Notably, CotW has been assigned to the outermost regions of the coat, possibly the crust (Kim *et al*., 2006; McKenney *et al*., 2010). Using AFM, Plomp *et al*. similarly observed long fibrous structures (rodlets) in a layer of *B. subtilis* spores (Plomp *et al*., 2005b). The position of this rodlet layer external to the ‘honeycomb’ layer has been modelled by Plomp *et al*. (2014). Whether or not these observations have any direct correspondence with our *ex vivo* fibres is a question for the future.

CotV–GFP failed to localise on spores of both *cotW* and *cotY* mutants (Imamura *et al*., 2011); this is consistent with a model where CotV is dependent on CotW for assembly of fibres and where CotY forms an ordered platform for these. This platform could be a hexagonally packed layer of CotY, equivalent to the ‘honeycomb’ layer described by Plomp *et al*. *(* 2014). It is clear that spores can incorporate fibrous elements in their architectural plan, and we have demonstrated that in addition to SpoIVA (Ramamurthi and Losick, 2008), fibres can indeed be constructed through self‐organisation of other coat proteins.

### Role of self‐assembly in spore coat construction

The proteins described here are a small fraction of those making up the spore coat. Nevertheless, we have shown that this subset of proteins has inherent properties of self‐organisation within a cytosolic environment; given the varied nature of these different proteins, it is unlikely that the *E. coli* host in which they were expressed could have specific machinery for higher‐order assembly of each one. An important emerging principle is that all the assemblies we have observed are one‐ and two‐dimensional; even the CotY stacks are essentially made up of two‐dimensional layers. These are just the type of stackable building blocks expected to make up the layered structure of the native spore coat. The relative positions of these layers have been modelled in Plomp *et al*. (2014).

Previous analyses had predicted both homotypic (e.g. CotY) and heterotypic interactions (e.g. CotV–CotW) among coat proteins; our results demonstrate a capacity for self‐organisation into supramolecular layered structures, that are consistent with these predicted interactions. However, we emphasise that the coat is biochemically complex and the effect that other coat components might have on forming higher‐order assemblies *in vivo* is unknown. It could be that the assemblies we have generated just appear as short‐range domains within the spore coat, with longer range structures being much more complex.

Evidence for the role of self‐organisation comes from the early work of Aronson and Fitz‐James (Aronson and Fitz‐James, 1971), who demonstrated partial reconstitution of outer coat layers of *B. cereus* that had been treated initially with DTT and urea; the presence of cystine was required for ordered layering, presumably facilitating disulfide exchange (Aronson and Fitz‐James, 1976). The involvement of intermolecular disulfide bonds in CotY oligomerisation has already been inferred (Zhang *et al*., 1993). CotY arrays required both heat and reducing conditions for complete disassembly. This strongly suggests that crystals are held together at least in part by exceptionally strong disulfide cross‐links. Other covalent cross‐links may also be involved (Kobayashi *et al*., 1996; Driks, 1999), but we did not test for these. Could recombinant CotY form disulfide cross‐links in the cytoplasm of the host cell, which is conventionally considered to be reducing; and by inference could such cross‐links be formed on the endospore within the native *B. subtilis* mother cell? The high degree of order and symmetry in the CotY crystal is likely to play a role here; the formation of multiple disulfide bonds as CotY subunits are recruited and precisely positioned into the growing lattice will be a highly cooperative process. An analogous effect can be seen in cooperative disulfide bond formation during protein folding (Chau and Nelson, 1992). Even if CotY lattice‐ordering was only a short‐range phenomenon in the native spore coat, such cooperativity could form an exquisite mechanism for ensuring exceptionally strong *intracellular* disulfide cross‐links. The additional functional advantage of cross‐linking in conferring thermal and mechanical stability (Fass, 2012) to the spore coat is clear. Indeed treatment of spores with reducing agents increases sensitivity to lysozyme and H_2_O_2_ in a number of species (Gould and Hitchins, 1963; Gould *et al*., 1970).

The general principles of cooperative self‐assembly and cross‐linking observed here may also be adopted in coat proteins of species of medical importance such as *Clostridium difficile*. Indeed, *C. difficile* has a number of cysteine‐rich coat proteins (Barra‐Carrasco *et al*., 2013; Paredes‐Sabja *et al*., 2014). Looking to future applications, the ability of *ex vivo* coat protein self‐assembly to achieve near perfect positional control at the molecular level could offer advantages in biologically inspired nanomanufacturing processes. The exceptional chemical and thermal stability, could confer additional advantages. It is also notable that a number of other biological surface structures are constructed out of thin layers of self‐assembling components such as the hydrophobins and chaplins of filamentous fungi and streptomyces (Gebbink *et al*., 2005) and bacterial S‐layers (Pum and Sleytr, 2014). These proteins also hold promise for biotechnological applications (Hektor and Scholtmeijer, 2005; Ekkers *et al*., 2014; Sleytr *et al*., 2014).

## Experimental procedures

### Bacterial expression strains, plasmids, media and growth conditions

*E. coli* strain MM294 (*endA1 hsdR17 supE44 thi‐1 recA1*) (Backman *et al*., 1976) and One Shot® TOP10 Electrocomp^TM^ *E. coli* (Invitrogen^TM^) were chosen hosts for maintenance of all plasmids. Recombinant His‐tagged proteins, CotY, CotE, CotW and the complex CotV–CotW were produced using expression plasmids based on pET28a or pETDuet expression vectors (Novagen, Merck Biosciences, Nottingham, UK NG9 2JR) described previously (Krajcikova *et al*., 2009; Müllerová *et al*., 2009). For CotV–CotW co‐expression, their genes were cloned into pETDuet‐1 plasmid designed for co‐expression of two target genes. The singly expressed proteins CotW, CotE and CotY were produced using expression vector pET28a. Three CotY constructs were made, one natural length (CotY), one with a hexahistidine C‐terminal tag (His_6_‐CotY) and one extended construct (His_6_‐CotYc). For the extended construct, cloning of the *cotY* gene into the pET28a plasmid introduced the following 19 amino acid residues in position 176–201 of the CotY C‐terminus: SIITMDKNSSSVDKLAAALEHHHHHH, while removing residues 176–181 KHHHNG. *E. coli* BL21(DE3)pLysS [*F* ^−^ *,ompT, hsdS_B_(r_B_* ^−^ *m_B_* ^−^ *), gal, dcm*, (DE3), pLysS, (Cam^R^) ] (Novagen) transformed with expression plasmids was cultivated in 50 ml Luria broth containing appropriate antibiotics at 37°C. For protein expression, the bacterial cultures were induced with 1 mM isopropyl β‐D‐1‐thiogalactopyranoside (IPTG) as cultures reached an OD_600_ of 0.6–0.7. The bacteria were grown for another 3 h and then harvested by centrifugation at 7000 *g* for 10 min.

### Purification of recombinant spore coat proteins

The recombinant spore coat proteins, His_6_‐CotYc, CotW and CotW–CotV were purified using metal‐affinity chromatography. Bacteria were resuspended in 800 μl of solubilisation buffer (25 mM Tris‐HCl, pH 8, 150 mM NaCl) and sonicated for a total of 100 s. After centrifugation of crude cell lysate at ∼39,000 *g* for 30 min, the supernatant was loaded onto a 1 ml Ni‐NTA agarose column and washed with 10 ml of washing buffer (40 mM imidazole in solubilisation buffer). The protein was then eluted with a step gradient of imidazole from 0.1 M to 1.0 M in solubilisation buffer – 1 ml of 0.1 M imidazole, 0.2 M imidazole, 0.3 M imidazole and 1.0 M imidazole; to improve the yield of soluble CotY protein, 8 M of urea was added to all buffers. Gel filtration was performed on a Superose‐6 10/300 GL column mounted on a FPLC system (GE Healthcare, Buckinghamshire, UK HP7 9NA). The column was equilibrated in solubilisation buffer. Of the sample, 0.5 ml was applied and eluted at a flow rate of 0.2 ml min^−1^. Eluted fractions were collected in aliquots of 0.5 ml. Standard MW markers used for the column calibration were thyroglobulin (669 kDa), apoferritin (443 kDa), beta‐amylase (200 kDa), alcohol dehydrogenase (150 kDa), albumin (66 kDa) and carbonic anhydrase (29 kDa). The void volume was calibrated with blue dextran.

### AFM

AFM measurements were performed on a PicoPSM 5500 (Agilent Technologies, Tempe, AZ, NB, USA) in a liquid cell filled with PBS buffer (1.8 mM KH_2_PO_4_, 10.1 mM Na_2_HPO_4_, 2.7 mM KCl, 140 mM NaCl, pH 7.5). AFM images were recorded in tapping mode using DNP‐S10 tips (Si_3_N_4_, Veeco, Camarillo, CA, USA) with a nominal spring constant of 0.06–0.58 N m^−1^.

### EM

Carbon‐coated copper grids were glow discharged in air and 5 μl of sample was applied for 1 min before blotting and staining in 0.75% (w/v) uranyl formate. Imaging was performed at a magnification of ∼ 52,000 on a Philips CM100 operating at 100 kV using a Gatan MultiScan 794 1Kx1K CCD camera (Gatan‐Pleasanton, CA, USA). Images were recorded at ∼400 nm underfocus.

### Isolation of macro‐assemblies of Cot proteins from *E* *. coli*

Hexahistidine‐tagged macro‐assemblies of Cot proteins were purified using a batch metal‐affinity method. Bacteria were suspended in 800 μl of solubilisation buffer and sonicated for a total of 100 s. One millilitre of NiNTA Agarose beads (Qiagen, Manchester, UK M15 6SH) was added to the cell lysate and incubated at 4°C for 20 min with shaking. After gentle centrifugation (< 500 *g*), unbound cell lysate was removed and NiNTA Agarose beads were resuspended in 5 ml washing buffer and washed. Protein was eluted and separated from NiNTA Agarose beads using 1M imidazole in solubilisation buffer and gentle centrifugation. Purified macro‐assemblies were pelleted by centrifugation at ∼ 39,000 *g* for 30 min and resuspended into 800 μl of solubilisation buffer.

We also used lysozyme treatment for more ‘gentle’ extraction of macro‐assemblies. Bacteria were resuspended in 1 ml of lysis buffer (50 mM Tris‐HCl), pH 7.5, 150 mM of NaCl, 5% glycerol (v/v), 1 mM of DTT, 1 mM of phenylmethanesulfonyl fluoride and incubated with lysozyme (300 μg ml^−1^) at 4°C for 4 h. To the cell suspension, 5 mM of MgCl_2_ and 1 μg ml^−1^ of DNase were then added and was then incubated at 4°C for 30 min.

Thrombin cleavage was carried out to remove the N‐terminal polyhistidine tags from pET28a vector based constructs. Ten microlitres of thrombin protease (GE Healthcare) was incubated with CotE for up to 6 h in 20 mM of Tris‐HCl, 150 mM of NaCl, pH 8.0 at room temperature.

Disassembly of CotY macro‐assemblies was assayed by incubation in reducing conditions of 8 M of urea solubilisation buffer with combinations of 99°C heat and 50 mM of DTT for 20 min. Multimers were separated using a 4–12% NuPAGE gel (Life Technologies) and detected using an anti‐polyHistidine antibody (Sigma‐Aldrich, Gillingham, UK SP8 4XT) through Western blot analysis.

### Preparation of thin sections

Cells expressing Cot proteins were pre‐fixed using 3% glutaraldehyde in 0.1 M phosphate buffer (pH × 7.0) overnight at 4°C. Secondary fixation was carried out using 2% aqueous osmium tetroxoide for 2 h at 25°C. Samples were dehydrated in an ethanol series and embedded in Araldite resin for 72 h at 60°C. Ultrathin sections of 70–90 nm in thickness were cut using a Reichert Ultracut E ultramicrotome and stained with 3% uranyl acetate for 25 min followed by Reynold's lead citrate for 5 min.

### Image analysis

For His_6_‐CotYc particles, all alignments and MSA were carried out using the IMAGIC‐5 software package (Image Science Software GmbH, Berlin, Germany) (van Heel *et al*., 1996). After direct alignment to the total average and cycles of MRA to selected averages, ∼2300 particles were selected from 55 images. Ten class averages were initially selected, which after refinement, were reduced to seven stable class averages. No symmetry was enforced.

Images of CotV–CotW filaments were analysed within the IMAGIC‐5 software package. Straight sections of the fibrous assemblies were boxed out. The resulting boxed images were normalised and then one of them was masked, filtered and used as an initial reference for a cycle of direct alignment. The total average of the aligned images was masked, filtered and used as a reference for a second cycle of direct alignment. After four cycles of alignment, images of fibres that aligned poorly were removed and another four cycles of direct alignment with tighter filtering of the references produced the final average image. One hundred thirty‐seven images of individual fibre segments remained for the final averaging, out of the 180 initially selected.

Images of crystals of His_6_‐CotYc were processed using the *2dx* software suite (Gipson *et al*., 2007). For data merging, we used the MRC suite of programmes (Crowther *et al*., 1996). Phase origins for individual images were refined against each other using ORIGTILTD, sequentially adding images of higher and higher tilt to the refinement. Initial estimates of tilt angle were made from highly tilted members of a series using EMTILT (Shaw and Hills, 1981). The common phase origin was found by comparing the phases of the reflections on each image within a z* window of 0.007 Å^−1^ to those of all the other images. At least two cycles of refinement of the phase origin, tilt angle and tilt axis for each image, were performed. The program LATLINE (Agard, 1983) was used to determine interpolated amplitudes and phases on a regular lattice of 1/300 Å^−1^ in z* by a weighted, least squares method for data up to 1/14 Å^−1^ resolution. A real‐space envelope of approximately twice the estimated crystal thickness (120 Å) was applied as a constraint. The output interpolated lattice lines were used as references for two cycles of crystal tilt and phase origin refinement. The variation of amplitude and phase along 0,0,*l* was estimated from the maximum contrast on each z‐section (Amos *et al*., 1982). Density maps were calculated within the CCP4 suite of crystallography programs (Collaborative Computational Project, Number 4, 1994). Three‐dimensional surface representations were rendered with CHIMERA (Pettersen *et al*., 2004).

## Supporting information

Supporting Information
