# Supplementary material for: Diverse supramolecular structures formed by self‐assembling proteins of the B acillus subtilis spore coat
Source: Mol Microbiol. 2015 May 15;97(2):347–59. doi: 10.1111/mmi.13030 (PMC4950064; doi:10.1111/mmi.13030)
Supplement: Supplementary file 1 — Supporting Information [file MMI-97-347-s001.zip › MMI_13030_supp-0001-Jiang_suppl_160315.pdf]

Diverse supramolecular structures formed by self-assembling proteins of the *Bacillus subtilis* spore coat

Shuo Jiang[1†], Wan Qiang[1†], Daniela Krajcikova[2†], Jilin Tang[3], Svetomir B. Tzokov[1], Imrich Barak[2], Per A. Bullough[1]

[1]Krebs Institute for Biomolecular Research, Department of Molecular Biology and Biotechnology, University of Sheffield, Sheffield S10 2TN, United Kingdom. [2]Institute of Molecular Biology, Slovak Academy of Sciences, Dubravska cesta 21, 845 51 Bratislava, Slovakia. [3]State Key Laboratory of Electroanalytical Chemistry, Changchun Institute of Applied Chemistry, Chinese Academy of Sciences, Changchun 130022, People's Republic of China. [†]These authors contributed equally to this work. Correspondence and requests for materials should be addressed to P.A.B. (email: p.bullough@sheffield.ac.uk)

Supplementary Table 1. The internal phase residuals determined after the imposition of all allowed two-sided plane groups calculated from one of the micrographs of His$_6$-CotYc crystals.

Two sided plane group	Phase residual (°) (90° random)	Number of Comparisons	Target residual based on statistics taking Friedel weight into account (°)
*p*1	21.3	40	
*p*2	45.2	20	30.8
*p*3	9.5*	34	21.3
*p*312	28.0	78	22.0
*p*321	18.2*	80	22.3
*p*6	30.1	88	23.5
p622	33.7	178	22.4

Internal phase residuals were determined from spots of IQ1-1Q5 to 20 Å resolution. The values marked with * are acceptable candidates for the symmetry as the experimental phase residual is better than that expected, based on the signal-to-noise ratio.

Supplementary Table 2. The internal phase residuals determined after the imposition of all allowed two-sided plane groups calculated from one of the micrographs of His_6-CotY crystals.

Two sided plane group	Phase residual versus with other spots (90° random)	Number of Comparisons	Target residual based on statistics taking Friedel weight into account
*p*1	17.1	38	
*p*2	11.1*	19	24.6
*p*3	5.4*	30	17.1
*p*312	11.4*	62	17.8
*p*321	10.8*	63	17.9
*p*6	7.5*	79	18.9
p622	11.5*	144	18.1

Internal phase residuals were determined from spots of IQ1-1Q5 to 20 Å resolution. The values marked with * are acceptable candidates for the symmetry as the experimental phase residual is better than that expected based on the signal-to-noise ratio.

Supplementary Fig. 1. Amino acid sequence alignment of *B. subtilis* polyhistidine-tagged CotY and its extended form.

```
                 1        10        20        30        40        50        60        70        80        90        102
  His6-CotY (1) GSSHHHHHHSSGLVPRGSHMSCGKTHGRHENCVCDAVEKILAEQEAVEEQCPTGCYTNLLNPTIAGKDTIPFLVFDKKGGLFSTFGNVGGFVDDMQCFESIF
 His6-CotYc (1) GSSHHHHHHSSGLVPRGSHMSCGKTHGRHENCVCDAVEKILAEQEAVEEQCPTGCYTNLLNPTIAGKDTIPFLVFDKKGGLFSTFGNVGGFVDDMQCFESIF
  Consensus (1) GSSHHHHHHSSGLVPRGSHMSCGKTHGRHENCVCDAVEKILAEQEAVEEQCPTGCYTNLLNPTIAGKDTIPFLVFDKKGGLFSTFGNVGGFVDDMQCFESIF

                 101      110       120       130       140       150       160       170       180       190        202
  His6-CotY (101) IFFRVEKLCDCCATLSILRPVDVKGDTLSVCHPCDPDFFGLEKTDFCIEVDLGCFCAIQCLSPELVDRTSPHKDK------------------KHHHNG--
 His6-CotYc (101) IFFRVEKLCDCCATLSILRPVDVKGDTLSVCHPCDPDFFGLEKTDFCIEVDLGCFCAIQCLSPELVDRTSPHKDKSIITMDKNSSSVDKLAALEHHHHHH-
  Consensus (101) IFFRVEKLCDCCATLSILRPVDVKGDTLSVCHPCDPDFFGLEKTDFCIEVDLGCFCAIQCLSPELVDRTSPHKDK                   HHH
```

His_6-CotY is the recombinant protein; His_6-CotYc is the C-terminally extended form of the recombinant protein.

Supplementary Fig. 2. SDS-PAGE of purified His$_6$-CotY from *E. coli* overexpression.

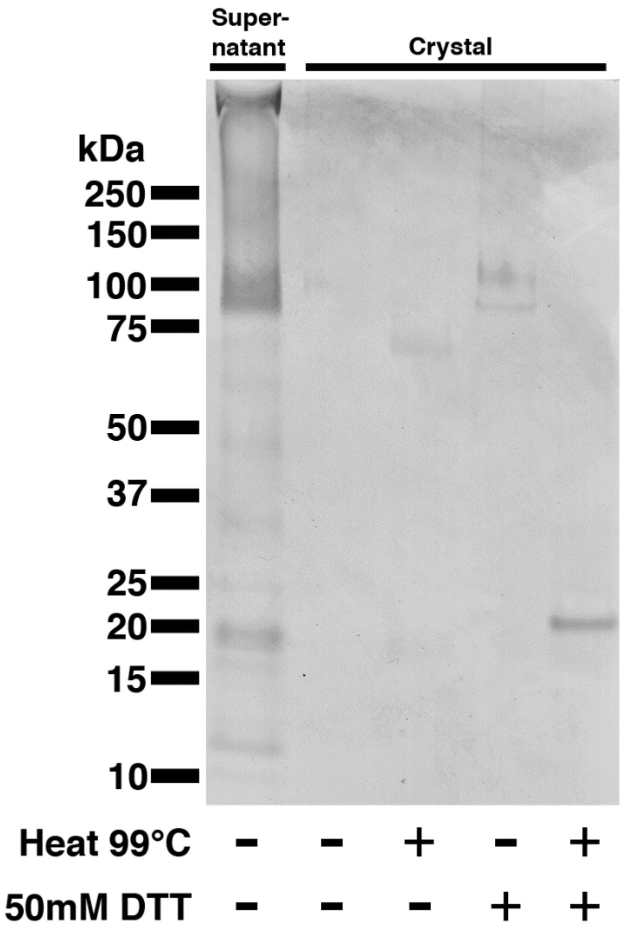

SDS-PAGE analysis of purified His$_6$-CotY crystal and supernatant fraction from *E. coli* overexpression. Lane 1 contains the nickel batch affinity purified supernatant fraction. Lanes 2-5 show nickel-affinity batch purified CotY crystals treated with combinations of denaturing conditions: heating at 99°C and 50mM DTT over 20 minutes. See Fig. 2 of the main text for more details.

Supplementary Fig. 3. Amplitude and phase variation along z* for selected *h*, *k* values for His_6-CotYc crystals. *p*3 symmetry was imposed.

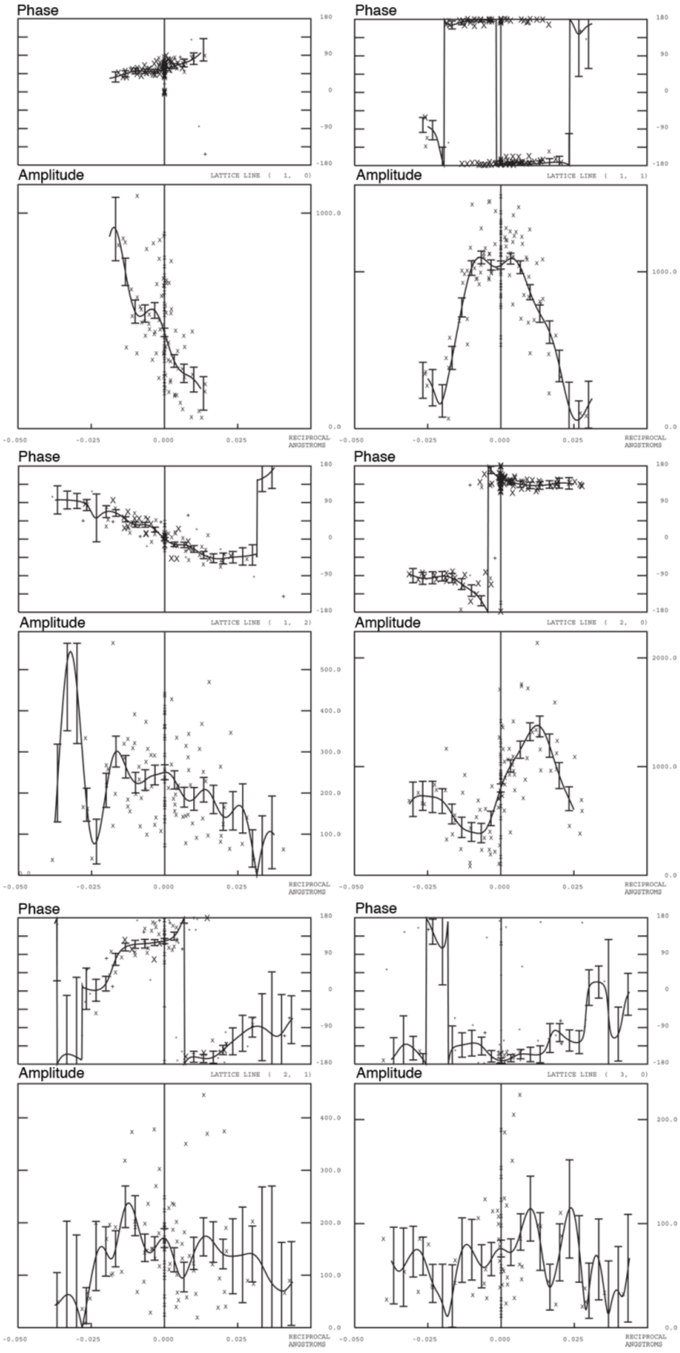

Lattice lines showing the phase variation along the z* axis in ° (top panels) and amplitude variation in arbitrary units (lower panels). Horizontal axis

displays distance from the origin of the lattice line. Standard error of fitted amplitude and phase values is represented in the error bars.

Supplementary Fig. 4. SDS-PAGE of co-expressed CotV-CotW purified by nickel affinity chromatography

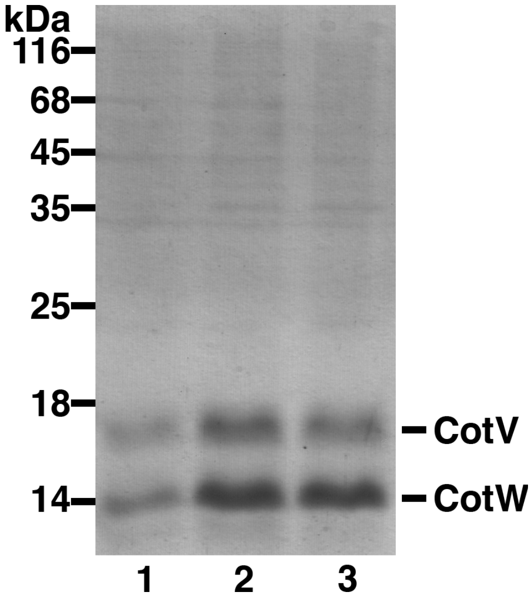

CotV-CotW eluted from nickel affinity column at increasing imidazole concentrations; 200mM imidazole (lane 1), 300mM imidazole (lane 2) and 1M imidazole (lane 3).

Supplementary Fig. 5. SDS-PAGE of purified His$_6$-CotE from *E. coli* overexpression.

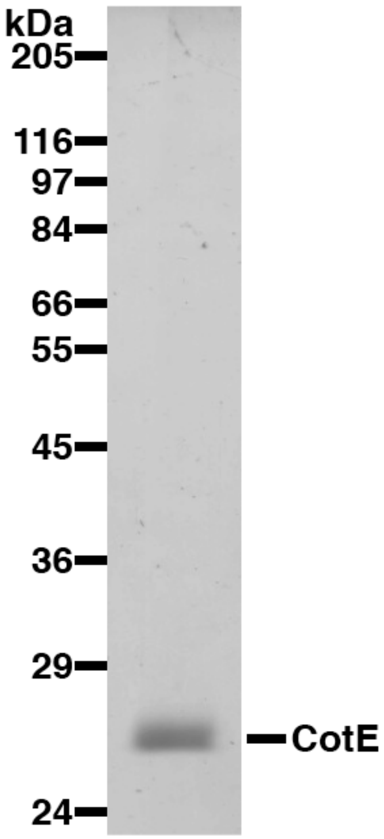

Lane shows SDS page of nickel affinity purified CotE in absence of DTT or heat.
